# How older people enact care involvement during transition from hospital to home: A systematic review and model

**DOI:** 10.1111/hex.12930

**Published:** 2019-07-13

**Authors:** Jenni Murray, Natasha Hardicre, Yvonne Birks, Jane O’Hara, Rebecca Lawton

**Affiliations:** ^1^ Yorkshire Quality and Safety Research Group, Bradford Institute for Health Research, Temple Bank House Bradford Royal Infirmary Bradford UK; ^2^ Social Policy Research Unit University of York York UK; ^3^ Leeds Institute of Medical Education University of Leeds Leeds UK; ^4^ School of Psychology University of Leeds Leeds UK

**Keywords:** involvement, model, older people, transitions

## Abstract

**Background:**

Current models of patient‐enacted involvement do not capture the nuanced dynamic and interactional nature of involvement in care. This is important for the development of flexible interventions that can support patients to ‘reach‐in’ to complex health‐care systems.

**Objective:**

To develop a dynamic and interactional model of patient‐enacted involvement in care.

**Search strategy:**

Electronic search strategy run in five databases and adapted to run in an Internet search engine supplemented with searching of reference lists and forward citations.

**Inclusion criteria:**

Qualitative empirical published reports of older people's experiences of care transitions from hospital to home.

**Data extraction and synthesis:**

Reported findings meeting our definition of involvement in care initially coded into an existing framework. Progression from deductive to inductive coding leads to the development of a new framework and thereafter a model representing changing states of involvement.

**Main results:**

Patients and caregivers occupy and move through multiple states of involvement in response to perceived interactions with health‐care professionals as they attempt to resolve health‐ and well‐being‐related goals. ‘Non‐involvement’, ‘information‐acting’, ‘challenging and chasing’ and ‘autonomous‐acting’ were the main states of involvement. Feeling uninvolved as a consequence of perceived exclusion leads patients to act autonomously, creating the potential to cause harm.

**Discussion and conclusion:**

The model suggests that involvement is highly challenging for older people during care transitions. Going forward, interventions which seek to support patient involvement should attempt to address the dynamic states of involvement and their mediating factors.

## 1 INTRODUCTION

There is long‐standing consensus, reinforced by policy‐led initiatives, that being involved in one's health care promotes choice and equity and indeed is an individual's right.^1^, ^2^ Involvement is seen as an essential tenet for improving both the quality and safety of care.^3^ Despite an articulation of a moral obligation and belief in the mechanisms by which involvement in care contributes to better health outcomes,^4^, ^5^ there is no clear understanding of what being ‘*involved in one's own care’* actually looks like. Various bodies of the literature talk *around* involvement with reference to the importance of the patient‐professional relationship^6^, ^7^ and influencing contextual factors such as having time and information^8^ but the more nuanced interactional and the dynamic nature of involvement has been largely overlooked. This is highly important in the current health‐care climate where there is increasing expectation that patients can, and increasingly more interventions that support patients to, take on more responsibility for their own care needs and decisions about treatment.^9^, ^10^, ^11^ Taking on responsibility necessarily involves patients performing ‘work’^12^ that includes reaching in to a complex health‐care system. 13, 14 The dynamics of how patients undertake this work may be a key determinant in the success or failure of these policy‐led initiatives and interventions.

Older people represent a particularly vulnerable group for whom involvement may be most challenging.^15^, ^16^, ^17^ They have complex health‐care needs, frequent hospital stays and high rates of readmission.^18^, ^19^ The transitional period from hospital to home, in particular, represents a fragile time for older people. Deconditioned from their hospital stay, they are often required to take on new care regimens alongside re‐integrating and coping at home. The individual experiences of older people during this period have been captured in numerous qualitative studies^20^, ^21^, ^22^, ^23^ but a synthesis that draws out and provides conceptual clarity about how people enact involvement has yet to be performed. This could, among other things, support the development of interventions.

We therefore sought to systematically review published qualitative data to provide greater conceptual clarity about the dynamic and interactional aspects of how patients enact involvement in their own care. Using the lens of older people transitioning from hospital to home, the overarching aim of the current study was to develop a model of patient involvement in care.

## 2 METHODS

A systematic and empirically driven approach to synthesizing the current evidence was employed to ensure that the concept of involvement was true to the patient experience. For the purposes of this review, involvement in one's own health care was defined as any actions undertaken, as well as thoughts and feelings held in support of pursuing a health‐ and well‐being‐related goal.

### 2.1 Study identification

Search methods aimed to identify qualitative studies reporting older people's experiences of transitioning from hospital to home. A search was run in MEDLINE, EMBASE, PsycINFO, Cumulative Index to Nursing and Allied Health Literature (CINAHL) and ProQuest to identify peer‐reviewed publications published between January 2005 and mid‐April 2019. This was a pragmatic choice aimed at identifying studies that reflect current service pressures and configurations (see Appendix S1 for a full list of search terms). A Google Scholar search employing the key search terms was used to supplement the search. Reference lists were searched and forward citation searching conducted. Included studies were as follows: empirical and qualitative; published in English language in peer‐reviewed journals; had study populations with a mean age exceeding 60 years; primarily included patients or informal caregivers; and focused on patient experiences of care transitions from hospital to home. Studies were excluded if they were linked to intervention studies to ensure that experiences represented usual care; focused on the general hospital experience rather than the experience of transferring from hospital to home; exclusively about the experiences of those going to nursing/residential homes or rehabilitation centres; or focused on one condition such as stroke to ensure that a range of experiences was explored.

### 2.2 Data extraction, analysis and quality assessment

Each paper was read, and findings about involvement, as per our definition, were initially coded (independently by JM and NH) using an existing involvement taxonomy as a theoretical framework (Appendix S2).^1^ This framework was chosen over others^24^, ^25^ as it provided greater conceptual clarity about different types of involvement at the individual level. Data relating to context (barriers and facilitators to involvement) and inferred consequences in relation to these findings were recorded. As coding progressed, we moved from a deductive to an inductive approach to capture aspects of involvement that did not fit into the theoretical framework. We checked that our interpretations of the findings aligned with each other and with the emergent categories (termed ‘types’ of involvement). Where required, we revisited the original paper to explore meanings and potential assumptions. To construct the model, we examined findings that reported multiple ways of enacting involvement, to understand how involvement could change within individuals in the context of one care episode or activity. To ensure that the model accurately represented the original data, the extracted findings from the studies were revisited and compared with the model. The model was subsequently interpreted to provide an overall understanding of the process of involvement of older people during transitions.

### 2.3 Patient and public involvement (PPI)

We convened a PPI session with six members of our existing panel patient to explore how they interpreted a selection of extracted quotes from the included studies. The group comprised older people (aged 70 and over) and two of their carers, all with experience of emergency hospital admission and discharges within the previous three years. The group sorted the provided quotes initially into ‘involved’ and ‘not involved’ and then into our suggested subtypes. Their sorting agreed with ours and the types of involvement, as defined by our research process, very much resonated with their experiences.

### 2.4 Quality assessment

The quality of included studies was assessed using an adapted version of the consolidated criteria for reporting qualitative research (COREQ).^26^, ^27^ The tool consists of 30 items (with a total possible score of 60) covering the research team and reflexivity, study design, setting and data collection, data analysis, findings and ethics. Two researchers (JM and NH), independently screened the studies and discrepancies, were resolved by discussion and revisiting the papers. Studies were not excluded on the basis of this assessment.

The protocol for this study is registered with PROSPERO No. CRD42017058696.

## 3 FINDINGS

**Figure 1:**
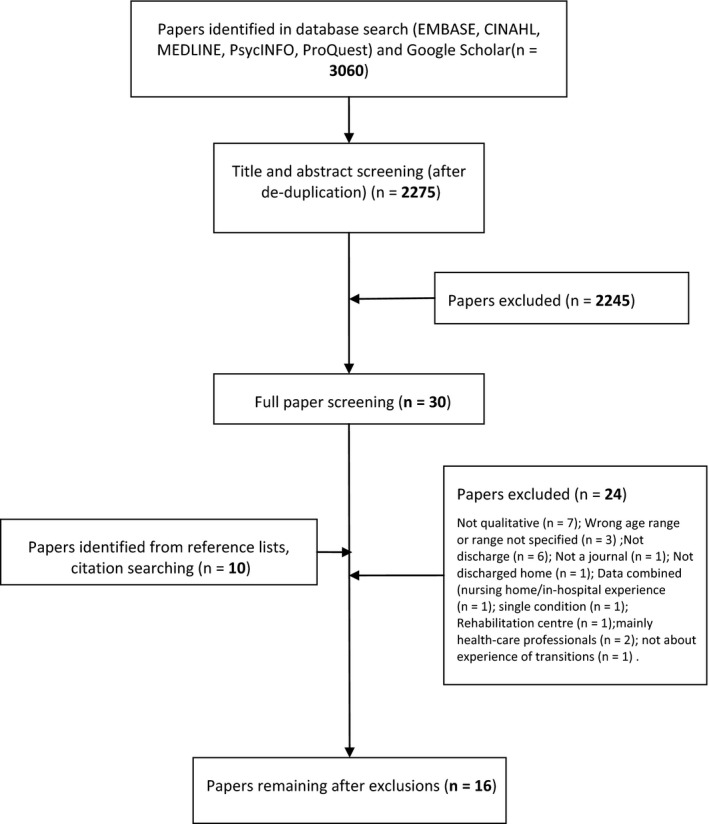
PRISMA Flow chart

Three thousand and sixty publications were identified, which through screening (Figure 1) provided sixteen studies that contributed to the development of the model. 20, 21, 22, 28, 29, 30, 31, 32, 33, 34, 35, 36, 37, 38, 39, 40 The studies collectively included 303 participants with 170 patients in 12 studies and 133 caregivers in eleven (see Table 1). One study specifically sought to include patients from ethnic minority backgrounds including gypsy travellers^30^; other studies did not specify the make‐up of their study population. A broad range of admitting conditions were reported across the studies, and the types and extent of information on social support for patients varied greatly. The experience of involvement in transitions was a specific focus within four studies.^29^, ^30^, ^34^, ^40^ The remaining studies were concerned with general experiences of care and transitions. Of note were three studies that, despite aiming to explore the general experience of transitions, reported extensively on involvement.^34^, ^38^, ^39^

**Table 1:** Study characteristics

Country	Participants (Gender F:M)	Ethnicity	Mean age (range)	Main reasons for Hospitalization	Data collection	Analysis	Main focus
Canada^29^	C = 18 (9F:9M)	N/R	77.4 (65‐89)	Deconditioning; hip fracture; hip/knee replacement; stroke.	Interviews and observations (pre‐ and post‐PD up to 6 wk	Grounded theory	Caregiver experience of transition and associated care processes
USA^20^	Pts = 9 (all M) C = 9 (all F)	All Caucasian	Pts (70‐88)	Hip/knee replacement; laminectomy; diabetes; arthritis; CHD; hypertension; alcohol abuse	3 × interviews up to 2 mo PD	Grounded theory	Patient and caregiver experience of transitions
Italy^37^	C = 18 (15F:3M)	N/R	48 (32‐80)	Neurological; orthopaedic; surgical NOS.	Single in‐depth interview mean of 11 d pre‐discharge. FGs at around 35 d PD	Grounded theory	Caregiver experience of transitions
Ireland^35^	Pts = 11 (5F:6M)	N/R	81 (71‐92)	N/R	Single interview within 2 wk PD	Phenomenology	Patient experience of transitions
Sweden^39^	Pts = 17 (7F; 10M) C = 5^a^	N/R	Pts 79 (65‐91)	Heart problems; infection; Rheumatic disease; Intestinal problems; Dehydration, Fracture; Pneumonia; Stroke; Intoxication.	Single interview up to 8 wk PD	Grounded theory	Patient and caregiver experience of transition
UK^34^	Pts = 7 C = 12 (whole sample 14F:5M)		Pts median 84 (75‐100)	Large range of medications does not state main discharge diagnosis	Single interview approximately 1 mo PD and/or week‐long medication diary	Thematic analysis	Patient and caregiver experience of hospital discharge relating to organization and management of medicines
Norway^33^	Cs = 11 (8F:1M;2 unknown)	N/R	N/R	Non‐specific	Single interview	Phenomenology	Caregiver experience of transitions
Denmark^28^	Pts = 14 (7F:7M)	N/R	80	Acute medicine	Single interview, 1 wk PD	Interpretive description	Patient experience of life in immediate PD period
Norway^32^	Pts = 7 (5F:2M)	N/R	70+	Acute disease or exacerbation of chronic illness	Single interview	Phenomenology	Patient experience of transitions
Canada^22^	C = 12 (7F: M = 5)	N/R	59	Hip fracture; stroke.	Single interview within 6 mo PD	Grounded theory	Caregiver experience of transitions
Sweden^38^	Pts = 14 (9F:5M)	N/R	88	Falls; infection; bowel problems; cancer; wrong medication; stroke; pneumonia.	Single interview 1‐2 wk PD	Content analysis	Patient experience of care transitions
Denmark^36^	Pts = 17 (F = 7, M = 10) C = 19 (11F:8M)	N/R	79 (70‐89)	CHF; stroke; COPD; pneumonia	Single interview 2‐5 wk PD	Grounded theory	Patient and caregiver experience of the organization and coordination of transitions
UK^40^	Pts = 23 (12F:11M)	N/R	82	N/R but patients on medical, renal and stroke wards	Single interview pre‐discharge	Phenomenology	Patient perceptions of effects of delayed transitions, involvement in planning and future care needs
UK^30^	Pts = 20 C = 4 (9F:15M)	Asian; black; gypsy traveller	Pts (60‐79); C (42‐76)	N/R	Single interview within 6 mo PD	Framework	Patient (minority ethnic communities) experience of hospital discharge
UK^21^	Pts=12^a^ C = 6^a^	White British	66	Fractures; gallstones; UTI; chest infection.	Audio and written diaries with single interview 8 wk PD	Phenomenology	Patient (non‐medically complex) experience of transitional care
Australia^31^	Pts = 19^a^ C = 19^a^	N/R	80	N/R	Three interviews: on admission, prior to discharge and 1 mo PD	Thematic analysis	Patient and caregiver experience of transitions

Abbreviations: C, caregiver; CHF, congestive heart failure; COPD, chronic obstructive pulmonary disease; F, female; M, male; N/R, not reported; NOS, no otherwise specified; PD, post‐discharge; Pt, patient; UTI, urinary tract infection.

a Gender not reported.

### 3.1 Study quality

All studies met more than half of the 30 quality assessment reporting criteria. Studies scored least well in relation to reporting about the research team, reflexivity and some aspects of the research design, scoring better on areas such as data analysis, findings and ethics.

### 3.2 Summary of types and subtypes of involvement

Four types and 12 subtypes of patient‐determined involvement were identified (Table 2). We also identified three types and seven subtypes of professionally mediated patient involvement along with a number of other contextual factors that appeared to influence involvement including, for example, having a supportive family and experiencing emotional problems.

**Table 2:** Identified types and subtypes of patient‐ and caregiver‐determined involvement

Patient‐determined types of involvement	Subtypes (references)	Description	Example (extracted quote or author's summary)^(reference)^
Non‐involvement	Desired^31^, ^37^, ^38^, ^45^	Explicit choice not to be involved through handing over responsibility for decision making and care to others	Patient was asked what kinds of medication she took and she replied ‘*No, that is for the nurse. I do not really use my head for that at all.’* 45
	Resigned^39^, ^45^	Not a choice but efforts to be involved are sensed as futile leading to a doing nothingness, apathy and abandonment	*‘I am so low now that I don't know what I can do. It's up to them now to try and sort it out. I can't see any way out of it’* 39
	Compliant^20^, ^22^, ^31^, ^33^, ^35^, ^37^, ^45^	Continuing with care plans despite having doubts and without questioning	Neither the patient nor his caregiver had any idea how long he should continue (using the wedge) once he got home. The patient continued to lie on his back because of the wedge which prevented healing of a bedsore acquired during a hospital stay^20^
	Complicit^31^, ^38^, ^39^	Justifying non‐involvement by comparing selves to others considered less fortunate or by putting complete unquestioning trust in staff	‘*I got no information about the operation or advice on how to behave afterwards. However, I think it was a simple operation, and the doctors are very clever, so I'm thankful for the job they did*’^31^
	Reluctant^22^, ^29^, ^31^	Dissatisfaction that involvement did not happen as envisaged with potential covert plans to seek alternative ways to be involved in care	Several caregivers expressed their discontent with the lack of information they received to prepare for their new care responsibilities^22^
Information‐Acting	Passively receptive/seeking^22^, ^29^, ^32^, ^33^, ^37^	Willingness to receive and give information that may be unexpressed or acted out through waiting for the ‘right time’ (with potential health consequences)	*‘I would just love to be informed….’* 32
	Actively seeking/giving^20^, ^22^, ^29^, ^32^, ^33^, ^36^, ^38^, ^39^	Taking or creating opportunities to ask questions. Most often in response to perceived failures in care delivery such as absent information	*‘We were pulling it (looking for information) on our own because otherwise it was just a black hole…you're kind of thirsting for information that whole time’* 22
Challenging and Chasing	No subtypes^22^, ^30^, ^32^, ^33^, ^38^	Challenging decisions that fail to take their wishes into consideration or chasing support when services are unresponsive	‘We rang up several times on the ward but they don't bother to answer or anything. Then two o'clock in the morning I rang up, I said *“What's happening, why can't you inquire more,”*’ The caregiver subsequently found out that his wife had been moved to intensive care^30^
Autonomous‐acting	Undesired^32^, ^33^, ^35^	Actions taken by caregivers and patients through being made responsible for care, without evidence that this was a desired role	*‘It's even more daunting and then I mean you have to juggle with the chemist and the repeat prescriptions and goodness knows what’* 33
	Necessity versus choice^20^, ^28^, ^29^, ^32^, ^35^	Essential actions carried out in the absence of any other perceived choice. More defined than ‘role’	A caregiver considering building their own ramp so that they could take their relative to essential medical appointments^35^
	Intentional^33^, ^38^	Planned enacting of care that differs to prescribed regimen	Altering a medication regime for convenience purposes^33^
	Unintentional^33^, ^38^	Unplanned enacting of care that differs to prescribed regimen	Inability to half a tablet meaning the patient took the whole one thus doubling the dose^33^
	Information management)^29^, ^33^	Ways of managing information without reference to choices or preferences	Patients developing self‐generated lists of medications that enabled them to receive, understand and check appropriate information^33^

### 3.3 Patient‐ and caregiver‐determined involvement

Types of involvement included ‘non‐involvement’, ‘information‐acting’, ‘challenging and chasing’ and ‘autonomous‐acting’. Non‐involvement represented a state in which people became passive recipients of care and even absent/failed care. The absence of patient and caregiver involvement was evident in all studies. Even where non‐involvement was ‘desired’, patients appeared to hold assumptions about the standards of care that they would receive. This was demonstrated through showing disappointment when expectations about care were not met.^32^ Resigned non‐involvement was reported alongside highly influential contextual factors such as low mood and ill‐health and was arguably the most debilitating subtype of non‐involvement.^28^, ^40^ The second type of involvement, information‐acting, could be active or passive. The literature showed that being more active often failed because health‐care professionals did not appear to ‘consider’ or understand expressed desires or know how to respond. 21, 37, 38, 39 This resulted in patients and caregivers moving between states of involvement in attempting to resolve a single aspect of care. Challenging and chasing, as the third type of involvement, highlighted the work and effort required to question staff and source information.^28^, ^30^, ^33^, ^34^ Examples of chasing were seen exclusively in caregivers. Challenging and chasing often came about through dissatisfaction, anxiety about the future and distrust of the system but was facilitated by interaction with a service that appeared willing to listen. The final type of involvement, autonomous‐acting, was often a consequence of non‐involvement in care, mediated through feeling excluded by professionals.

### 3.4 Professionally‐determined types of involvement

Patients and caregivers alluded to three ways in which they felt professionals mediated involvement through ‘exclusion’, ‘information‐seeking/information‐giving’ and ‘consultation’.

In general, patients and caregivers suggested that care providers hampered their efforts to obtain information. Being busy, appearing unapproachable and authoritarian, and being focused on discharge, meant that patients felt unable to pose questions. Patients suggested that professionals did not listen, avoided eye contact, demonstrated little insight into the family circumstances and did not see patients as individuals. Even where patients felt able to approach staff, nurses appeared unable to answer their questions, deferring to absent doctors.

Nobody tells me (about leaving hospital). I asked them (nurses) but they don’t even know themselves).^40^

Despite not always having the answers to questions, there was evidence that some staff did seek to obtain information to the extent of chasing. For example, Andreasen^28^ reported on how one member of staff *‘phoned God and everybody’* on the patient's behalf only to be told that they would have to wait until the following week for the essential item of toileting equipment.^28^ This could represent a form of staff exclusion from the services that they work in but also challenging and chasing, similar to that observed in patient‐determined involvement. A more extreme version of patient perceived exclusion was observed in two studies where health professionals appeared to ‘close the door’ by overtly declining requests for help.

I told them I couldn’t manage at home and needed to stay a few more days. But the doctor told me there was no place at all for me on the ward or in hospital.^39^

Staff also mediated involvement through information‐giving. While this could be useful, it could equally be unidirectional, lack consultation and tailoring, and be inappropriately timed. Patients did, however, indicate that information, if given in the right way, could encourage involvement. Finally, a more positive approach to encouraging involvement through professional consultation was described as including activities such as formal discharge planning meetings, home visits or more informal routes such as a bedside consultation approaches.^21^, ^29^, ^31^, ^38^

### 3.5 State‐change model of involvement

**Figure 2:**
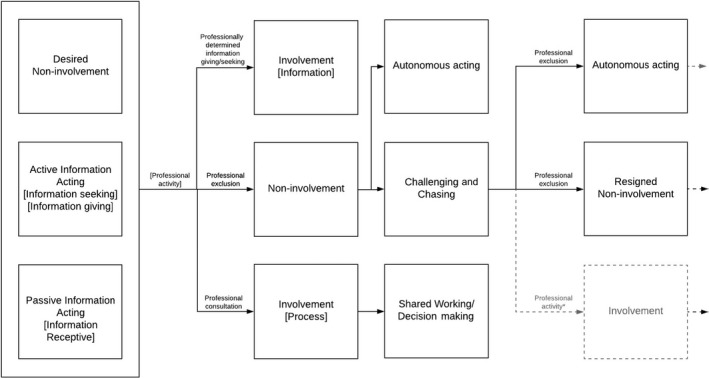
State‐change model of involvement. Dashed lines represent pathways within the state‐change model that were not reported in the current body of literature but are possible

By exploring findings which reported multiple types and subtypes of involvement, we were able to observe that the process of enacting care is not static or necessarily a trait‐determined approach. Rather, people change their ‘status’ depending on their interactions with services and other contextual factors. Thus, in constructing a model that represents this dynamic interactional process, we have oriented ‘types’ of involvement into ‘states’ (Figure 2).

Findings reported up to four state changes. The model commences with information‐acting and desired non‐involvement. Despite the fact that some findings started at the point of professional exclusion and even challenging and chasing, it is likely that patients and caregivers were at least passively information‐receptive at a prior point. Professionally mediated involvement influenced the next step of patient‐determined involvement, where exclusion, leading to non‐involvement, could move into a state of autonomous‐acting or challenging and chasing. In the literature, there were frequent examples of the final state of involvement as being autonomous‐acting and resigned non‐involvement brought about through feelings of being excluded. Positive outcomes, that is being involved as desired, were rarely observed but it is recognized that professional consultation at any point could result in involvement in either the process of care delivery (eg taking part in team meetings) or through being informed about care. Similarly, although shared decision making was not observed in the findings, professional consultation may support this. These final states (which are analogous to having information needs met) which were not observed, but are possible, have been included in the model with appropriate annotation. Some states appeared to be momentary, representing a thought, followed shortly after by a decided‐upon strategy. Given that this is based on patient recall, no information on the duration in which people occupied various states was available. Finally, the process of enacting involvement could continue beyond the model depicted here. So, for example, resigned non‐involvement may be transitory, moving on to another form of enacting involvement. Examples of changes in states are detailed below.

I tried to explain that it wouldn’t work (referring to technical aid) (CHALLENGING & CHASING), but they didn’t consider that (PROFESSIONAL EXCLUSION), then I thought I won’t argue (RESIGNED NON‐INVOLVEMENT), I won’t use it at home (AUTONOMOUS‐ACTING).^39^

The above example demonstrates how a state (ie resigned non‐involvement) might be momentary.

I told them my doubts and fears (INFORMATION‐ACTING: ACTIVE) but no‐one understood me (PROFESSIONAL EXCLUSION) and I felt like they were not going to tell me anything else. I realised I had to manage on my own (UNDESIRED‐AUTONOMY).^37^

## 4 DISCUSSION

The state‐change model of patient involvement clearly shows that enacting care is a dynamic, interactional and complex process, at least for older people during transitions. The model is based on patient recall of personal experiences of involvement and offers a much greater insight into what involvement looks like than previously published frameworks and taxonomies which have been largely based on imagined preferences.^1^, ^8^, ^25^ A clear message from the model is that involvement is not solely a trait, but a changeable way of being that is mediated by professional actions (as depicted in the model) and other contextual factors such as physical and cognitive abilities, emotions and social support mechanisms. It provides an understanding of the challenges to involvement beyond the consultation where most care is enacted and beyond managing care in the context of single long‐term conditions. Explicit in the model are the thoughts and feelings of patients during moments of attempting to enact care through, for example, feelings of exclusion and feeling resigned. This offers a personalized understanding of involvement. The model further demonstrates that movement between states is not always desirable and can be instinctual, occurring within moments. It shows that in the broader context of involvement, patients make ‘jumps’ across extremes of involvement; a movement previously thought conceivable but unlikely.^1^ Within the model, involvement is seen to be non‐linear with many processes leading back to non‐involvement but potentially equally able to change course at many points. Finally, contrary to an existing taxonomy,^1^ there are no ‘levels’ of involvement and no inferred hierarchy that culminates in a most desired state of autonomous decision making. ‘Autonomous‐acting’, in this model, was often a necessary undesired state.

Understanding involvement through the state‐change model has several important implications for care. The model suggests that failing to respond to patients’ attempts to be involved in their own care could have negative future consequences of varying proportionality (eg future distrust, safety errors, readmissions). The literature which informed the model, identified staff behaviours such as avoiding eye contact, as contributing to patient perceived exclusion but offered little insight into why this happened. The broader literature suggests that work pressures, difficulties in managing patients’ fears, anxieties and unrealistic expectations about their health and care all contribute to avoidant behaviours among health‐care staff.^41^ These barriers are likely to be further exacerbated by system pressures that prioritize patient flow^42^ to reduce ‘bed blocking’, particularly in relation to older people. Ironically, behaviours, which exclude patients from their care, promote autonomous‐acting so that people make independent judgements and sometimes take risky actions; the very activities that health‐care professionals are disinclined to support.^43^ Some of the autonomous actions observed in the current review were beneficial; however, a number resulted in or had the potential to cause harm.

The model presented a number of states of involvement that could be misconstrued by health‐care professionals. Passive information‐seeking and various types of non‐involvement (non‐complicit, compliant, reluctant and resigned) were pervasive states across studies and could suggest patient‐chosen disinterest or even full comprehension. For busy staff, these signals give permission for non‐interaction, with the concomitant risk that patients leave hospital with greater unmet needs and therefore increased risk of hospital readmission.^44^, ^45^ This is of particular concern for patients without caregivers who frequently enact ‘challenging and chasing’ on their behalf. Challenging and chasing is demanding, requiring individual capacity, and social and material resources: assets which many vulnerable people do not possess.^12^, ^13^ A system of care which leans towards a reliance on capacities to challenge and chase may thus fuel social inequalities in health.

### 4.1 Limitations

A number of limitations to this work have been identified. Individual studies reported predominantly negative experiences of patient involvement. This may simply reflect ‘reality’; however, they could also partly be an artefact of the methods. Observational methods to explore staff‐patient interactions were applied in only one study,^29^ and these could illuminate how staff communicate with patients. Learning from good care and understanding how health‐care professionals support involvement under challenging circumstances would contribute to the spread and adoption of sustainable approaches.^46^, ^47^

Findings did not necessarily report the conclusion of people's endeavours. This may be because the focus of many of the studies was on experience and not involvement per se*.* The model therefore attempts to represent what could be reasonable conclusions where professionally mediated involvement could result in successful patient and caregiver involvement. This would require testing in future research. A clear caveat is that acquiring an involved status does appear to take considerable ‘work’; capacity to undertake this work may be permanently or temporarily beyond the reach of many vulnerable older individuals.

Finally, the model only represents the experiences of older people with multimorbidity who did not have cognitive impairment or dementia. Neither does it represent those receiving specialist services such as cancer treatment nor condition‐specific self‐management support who may experience involvement differently. It is unclear how, or if the model, would need to be adapted to fit other patient groups, including those with dementia and younger people, who may have higher demands and expectations.^48^ This model clearly needs further testing to understand its general applicability; however, given the vulnerability of this particular group of patients, understanding how they are involved in their own care is worthy of specific study and theorizing.

### 4.2 Implications for research and practice

Interventions aiming to support older people to transition from hospital to home have been the subject of numerous systematic reviews.^49^, ^50^, ^51^, ^52^, ^53^ Self‐management and/or education, as a way of empowering individuals to be involved in and take control of their care, was the second most common component of these multicomponent interventions.^49^, ^50^, ^53^ The contribution of self‐management to outcomes is challenging to disentangle, but there is some suggestion that interventions which aim to ‘*enhance patient capacity to reliably access and enact post discharge care’* could be most effective (in terms of reducing hospital readmissions).^51^ Part of enhancing capacity to enact care could involve creating the space for patients to be heard in hospital through the application of good professional communication skills and good professional‐patient relationships built upon trust. In the wider literature, communication skills training is a recognized component of self‐management.^54^ Evidence of training, however, is not apparent in existing transition interventions for older people,^49^, ^50^, ^51^, ^52^, ^53^ and in the studies in the current review, use of such skills was not apparent. Reasons are likely to vary, but the hospital setting itself is likely to be a factor. System pressures emphasizing patient flow may limit the opportunities for relationship‐building. The ethos of hospital care is to manage acute illnesses rather than support maintenance of long‐term conditions. Establishing meaningful involvement with patients under these circumstances may be particularly challenging and resource intensive. There are current improvement drives towards greater ‘patient activation’^54^ in relation to facilitating involvement and self‐management, but these are at risk of labelling patients by trait and fail to acknowledge the dynamic nature of involvement that fluctuates in response to various compelling contextual factors. The ultimate aim of supporting involvement would be to create a space that enables patients to shift from passive information‐acting to actively voicing their concerns in such a way that does not fundamentally seek to change their way of being and that effectively meets people ‘on their own turf’.^55^

## 5 CONCLUSIONS

Previous studies reporting older people's experiences of involvement during hospital stays indicate that patients want to be involved in their care.^15^, ^56^, ^57^ This review and interactional model supports this and shows that non‐involvement is not a desired state for most patients but a consequence of system‐level forces and other contextual factors that act to erode efforts to become involved. Future interventions require a more nuanced approach that supports staff to recognize all states of patient involvement as valid, to reflect on how their behaviours can influence involvement and to understand how these can impact on patient safety and experience. For those who desire non‐involvement, a greater understanding of the factors that perpetuate this state will need to be explored. Respecting the wishes of these individuals while countering against the potential to widen health inequalities will be a fine balance for such interventions.

## CONFLICT OF INTEREST

There are no conflicts of interest.

## Data Availability

Data sharing is not applicable to this article as no new data were created or analyzed in this study.
