# Supplementary material for: How older people enact care involvement during transition from hospital to home: A systematic review and model
Source: Health Expect. 2019 Jul 13;22(5):883–93. doi: 10.1111/hex.12930 (PMC6803411; doi:10.1111/hex.12930)
Supplement: Supplementary file 1 [file HEX-22-883-s001.doc]

## Appendix 1. Thompson’s taxonomy of involvement^1^

| **Patient Desired level** | **Patient-determined of involvement** | **Co-determined** | **5 levels of care provider-determined involvement** |
| --- | --- | --- | --- |
| 4 | Autonomous decision making |  | Informed decision making |
| 3 |  | Shared decision-making | Professional -as-agent |
| 2 | Information giving |  | Consultation |
| 1 | Information seeking / receptive |  | Information giving |
| 0 | Non-involvement |  | Exclusion |
