# Supplementary material for: How older people enact care involvement during transition from hospital to home: A systematic review and model
Source: Health Expect. 2019 Jul 13;22(5):883–93. doi: 10.1111/hex.12930 (PMC6803411; doi:10.1111/hex.12930)
Supplement: Supplementary file 2 [file HEX-22-883-s002.doc]

## Appendix 2. Search methods

Search terms for Medline

1. Elderly OR Elder OR Old OR Geriatric OR Aged OR Mature OR Senior).ti,ab
2. (Discharg* OR Transition* OR Transfer OR Readmission).ti,ab)
3. (Experience OR Satisfaction OR Story OR Opinion).ti,ab)
4. AND (1-3)
5. [Human age groups Aged OR Aged,80 and over] [Languages English]

The terms were modified as require for other electronic databases

**Search words for Google and Google scholar**

Older people and transitions and experience (each word interchanged with elderly, discharge and qualitative)
